# A Quantum-Classical Hybrid Solution for Deep Anomaly Detection

**DOI:** 10.3390/e25030427

**Published:** 2023-02-27

**Authors:** Maida Wang, Anqi Huang, Yong Liu, Xuming Yi, Junjie Wu, Siqi Wang

**Affiliations:** 1 School of Mathematics and Statistics, Wuhan University, Wuhan 430072, China; 2 Institute for Quantum Information & State Key Laboratory of High Performance Computing, College of Computer Science and Technology, National University of Defense Technology, Changsha 410073, China

**Keywords:** deep learning, quantum machine learning, image anomaly detection, quantum hybrid deep neural network

## Abstract

Machine learning (ML) has achieved remarkable success in a wide range of applications. In recent ML research, deep anomaly detection (AD) has been a hot topic with the aim of discriminating among anomalous data with deep neural networks (DNNs). Notably, image AD is one of the most representative tasks in current deep AD research. ML’s interaction with quantum computing is giving rise to a heated topic named quantum machine learning (QML), which enjoys great prospects according to recent academic research. This paper attempts to address the image AD problem in a deep manner with a novel QML solution. Specifically, we design a quantum-classical hybrid DNN (QHDNN) that aims to learn directly from normal raw images to train a normality model and then exclude images that do not conform to this model as anomalies during its inference. To enable the QHDNN to perform satisfactorily in deep image AD, we explore multiple quantum layer architectures and design a VQC-based QHDNN solution. Extensive experiments were conducted on commonly used benchmarks to test the proposed QML solution, whose results demonstrate the feasibility of addressing deep image AD with QML. Importantly, the experimental results show that our quantum-classical hybrid solution can even yield superior performance to that of its classical counterpart when they share the same number of learnable parameters.

## 1. Introduction

Anomaly detection (AD) [1], which is also called one-class classification [2], is a task of identifying abnormal samples that differ from the given normal data. Against the background of industrialization and informatization, AD has important applications in many fields. For example, it can be used to monitor the operating states of machines or systems [3], detect whether systems are invaded [4], identify and detect financial fraud [5], perform out-of-distribution detection [6,7], and assist doctors with medical diagnosis [8]. The importance of AD has encouraged many researchers to develop effective AD algorithms. Due to the scarcity of anomalies, AD is often addressed as a task of semi-supervised/unsupervised learning. Its core idea is the assumption that the majority of accessible original data are normal, and the anomalous samples of data are not known a priori. Then, the goal is to build a mathematical model of normal data through training. When test data do not conform to the model, they are considered anomalous data. Up to now, there has been a large number of solutions for AD. For example, mainstream AD methods can be categorized into statistical methods, density-based methods, and distance-based methods (reviewed in Section 2.1). With the advent of deep learning and its great success in recent machine learning (ML) research, AD methods based on deep neural networks (DNN), which can be shortened to deep AD, have attracted much attention from the research community. Without a time-consuming feature engineering stage, deep AD makes it possible to learn a model directly from the raw input data, which can be more convenient and effective than the method of traditional AD. Therefore, such merits render deep AD particularly suitable for handling visual data, such as images, which have undergone explosive growth due to the popularization of digital devices. Thus, we focus on discussing image AD as a representative task for exploring deep AD in this paper.

Quantum computing refers to computing tasks or algorithms that can be accomplished by using quantum computers. With the rapid development of quantum technology, the tremendous potential of quantum computing makes this a key research direction in many countries. Considering the popularity of ML, quantum machine learning (QML), which aims to combine the advantages of both quantum computing and machine learning, naturally receives considerable interest. To exploit the existing ML foundation for QML research, a promising direction is that of extending classical ML methods to their quantum or quantum-classical hybrid counterparts. For instance, researchers have proposed QML models such as the quantum autoencoder [9,10,11], quantum Boltzmann machine [12,13], quantum generation antagonistic learning [14,15,16,17], and quantum kernel methods [18,19,20]. Similar to the success of DNNs in classical machine learning, quantum deep neural networks (QDNNs) and quantum-classical hybrid deep neural networks (QHDNNs) also show extraordinary prospects in the field of QML. Currently, there are numerous studies on QML, but most of them still focus on ordinary ML tasks, such as binary/multi-class classification. As far as we know, image AD has not been approached in a deep manner with a QML solution due to its more challenging nature. Therefore, this paper intends to fill this blank by exploring the feasibility and effectiveness of QML in deep image AD.

To this end, we present a QML solution for deep image AD within our knowledge. Our solution is built upon a QHDNN that is inspired by the classical deep AD solution, deep support vector data description (DSVDD) [21]. We explore various means of designing the QHDNN, and we conducted extensive experiments to test the proposed hybrid solution. Our results on public image benchmark datasets not only justify the feasibility and effectiveness of introducing a quantum module into a deep AD model, but also reveal that the proposed QHDNN can even outperform its counterpart in some cases.

## 2. Related Work

### 2.1. Anomaly Detection

In the field of ML, the concept of AD has been proposed for a long time. In the beginning, AD was often applied for the inspection of large industrial machines. In recent years, with the widespread use of computers, AD has enjoyed more and more applications in practice. In view of this situation, researchers have proposed many AD methods [22]. Early AD methods were based on statistical calculations. They used “normal data” to estimate probability density functions, such as in Parzen’s density estimate method [23]. If the density function of the test samples is below the threshold value, we could consider them as “abnormal data”. However, these algorithms often require a large number of samples to yield an accurate density estimation. Another representative AD method is the one-class SVM (OCSVM) [24]. The goal of OCSVM is to find the maximum margin hyperplane in the feature space to separate the data from the origin. Similarly, the classical support vector data description (SVDD) algorithm [25] aims to map the original normal data to a hypersphere that is as small as possible. If the test sample falls outside the hypersphere, it is considered abnormal. There are also other types of classical AD algorithms, such as the linear programming (LP) classifier, which has the purpose of minimizing the volume of a prism [26]. However, most of the classical AD methods lack the ability to learn useful features from the original data, which makes it hard for them to escape from the painful feature extraction process [21]. Since recent studies [27,28] have made tremendous progress in representation learning with DNNs, it is natural to approach AD with deep learning methods, which has motivated researchers to establish a new research branch named deep AD. One of the most prevalent deep AD models is the autoencoder [29], which contains a symmetric encoder-decoder structure for encoding raw input data. Nevertheless, they are not trained by an AD-based objective function and rely on heuristic methods based on reconstruction errors [21]. Thus, Lucas et al. proposed a deep AD solution named deep SVDD (DSVDD) [21], which was inspired by the classical AD method, SVDD. Specifically, DSVDD employs a DNN to map raw input data to a new feature space and encourages them to fall into a hypersphere with a minimum volume in this space. Following DSVDD, several deep AD methods were proposed, and they significantly improved the performance in tasks such as deep image AD. For instance, a major breakthrough in deep AD was achieved with a geometric-transformation-based method [30]. It transformed one-class data into multiple classes through geometric transformation to learn good representations and perform AD. Tack et al. [31] proposed the first contrastive-learning-based framework to further improve deep AD performance. In this paper, considering the simplicity and effectiveness, we chose DSVDD as the foundation for exploring the possibility of addressing deep image AD with QHDNN.

### 2.2. QML

In recent years, the field of QML has rapidly developed. Multiple QML algorithms have emerged, such as the quantum autoencoder [9,10,11], the quantum Boltzmann machine [12,13], quantum generative adversarial learning [14,15,16,17] and the quantum kernel method [18,19,20]. These quantum algorithms have been experimentally proven to be effective. In the research on QML, QHDNN is one of the promising directions. Unlike QML algorithms that are based on pure quantum circuits, the aim of QHDNN is to take advantage of both classical deep learning and promising quantum computing. Based on mature DNN structures, QHDNN enjoys easier implementation and better exploitation of existing DNNs’ power, which motivated a line of recent work. For example, Chen et al. [32] trained a QHDNN to classify two types of quantum data. However, these data types are not universally applicable. Wilson et al. [33] proposed an open-loop hybrid algorithm called quantum kitchen sinks (QKS) and used it to solve a binary classification problem for two handwritten numbers. Skolik et al. [34] used a QHDNN trained with a hierarchical learning strategy to perform handwritten number classification. Although these QHDNNs are feasible, their quantum structures are too specialized to be combined with other existing algorithms. The problem was not discussed until Mari et al. [35] proposed a QHDNN for transfer learning. Its dressed quantum circuit (DC) could be easily incorporated into existing neural network algorithms. At present, most QML solutions focus on several ordinary learning tasks, such as binary classification. As for AD, we also noticed that few recent works were devoted to using quantum circuits for AD. Concretely, Gunhee Park et al. [36] proposed a variational quantum one-class classifier; Alona Sakhnenko et al. [37] proposed a hybrid classical-quantum autoencoder for anomaly detection in tabular data. Nevertheless, to the best of our knowledge, no QML work has delved into deep image AD, despite its importance. Therefore, this paper intends to fill this gap by exploring the feasibility of QML in the deep image AD field.

## 3. A Quantum-Classical Hybrid Solution for Deep Anomaly Detection

As far as we know, the application of a QHDNN in deep image AD is still a research gap. To fill this gap, we intend to make an attempt by devising a novel quantum-classical hybrid solution. Since DSVDD has been proven to be an intuitive but effective baseline that pioneered the exploration of deep AD for images, we developed our quantum-classical hybrid solution based on DSVDD. In this section, we first introduce DSVDD, and then present our quantum-classical hybrid method.

### 3.1. DSVDD

**Figure 1:**
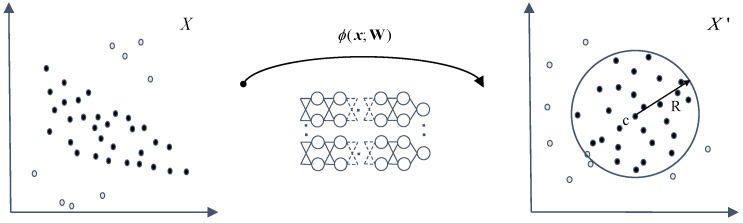
Conceptual diagram of DSVDD. DSVDD [21] is a classical deep AD solution that adopts a DNN to map “normal data” (represented by solid circles in the diagram) into the hypersphere.

DSVDD [21] is a classical deep AD solution whose conceptual diagram is shown in Figure 1. To be more specific, DSVDD employs a classical DNN to map raw data into a new feature space and let them fall into a hypersphere. The hypersphere is supposed to contain as many normal data as possible with a minimum volume (“normal data” refers to single-class data that have been given during training, while anomalies are considered to be unknown in AD during the training stage). Afterward, the training ends up with a learned hypersphere. Once the test data fall outside it, the data are considered abnormal. Assuming that the given training dataset is $X\subseteq R^{d}$, $\phi \left(\cdot ;W\right)$ is a DNN that maps the input dataset to the embedding set $X^{\prime }\subseteq R^{p}$ in the new feature space. The network has *J* layers, with a set of learnable parameters $W=\left\{W^{1},W^{2},\ldots ,W^{J}\right\}$, where $W^{j}$ are the parameters of layer $j\in \left\{1,\ldots ,J\right\}$. $\phi \left(x;W\right)\in X^{\prime }$ is the mapped representation of x∈X given by network ϕ with parameters W. The hypersphere in the new feature space is characterized by a radius of R>0 and the center $c\in X^{\prime }$. For the training dataset $X=\left\{x_{1},\ldots ,x_{n}\right\}$, the soft-boundary DSVDD loss function is defined as:

(1) $$\min_{R,W}\;R^{2}+\frac{1}{vn}\;\sum_{i=1}^{n}max\left\{0,{∥\phi \left(x_{i};W\right)\;-c∥}^{2}\;-\;R^{2}\right\}+\frac{\alpha }{2}\;\sum_{j=1}^{J}{∥W^{j}∥}_{F}^{2}.$$

As in DSVDD, the volume of the hypersphere is minimized by minimizing *R*. The second term is the penalty term for points falling outside the hypersphere. The hyperparameter $v\in \left(0,1\right]$ controls the trade-off between the sphere volume and violations of the boundary. The last term of (1) is the regularization term, which is used to prevent overfitting. α is the coefficient of the regularization term. With further simplification, the one-class DSVDD loss function is defined as:

(2) $$\min_{W}\;\frac{1}{n}\sum_{i=1}^{n}{∥\phi \left(x_{i};W\right)-c∥}^{2}+\frac{\alpha }{2}\sum_{j=1}^{J}{∥W^{j}∥}_{F}^{2}.$$

**Figure 2:**
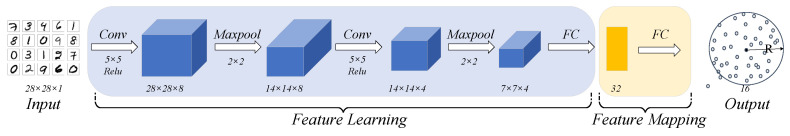
The DNN of DSVDD. It is composed of convolutional (Conv) layers, max-pooling (Maxpool) layers, and fully connected (FC) layers.

For the test data x∈X, we define the anomaly score according to the distance from the center of the hypersphere.

(3) $$s\left(x\right)={∥\phi \left(x;W^{*}\right)-c∥}^{2},$$

where $W^{*}$ are the network parameters of the trained model. In DSVDD, the whole process is divided into two steps. **Step 1:** pre-training. This is accomplished with a classical autoencoder, which has an encoder-decoder structure. For an input datum x, the encoder maps it to an embedding $x^{\prime }$, and the decoder aims to reconstruct the original x from $x^{\prime }$. In this process, the parameters W of the encoder are adjusted to reduce the reconstruction errors. To initialize the center of hypersphere c, we average all of the embeddings in $X^{\prime }$. After pre-training, the obtained encoder and the center c are preserved, while the decoder is discarded. In this way, we have actually obtained a preliminary hypersphere. **Step 2:** fine-tuning. In this step, we minimize the volume of the preliminary hypersphere according to the objective function in the objective function (1) or the objective function (2). In the objective function (1), the hypersphere is shrunk by directly penalizing the radius and the embeddings that fall outside the hypersphere. Differently, the objective function (2) contracts the hypersphere by reducing the average distance of all embeddings from the center. Finally, we can use Equation (3) to calculate the anomaly score of a given test data x. Therefore, we judge whether data are abnormal according to their scores. Since one-class DSVDD performed slightly better than its soft-boundary counterpart in image AD in the past [21], this paper mainly used one-class DSVDD as the baseline. Figure 2 shows the specific structure of DSVDD. We used the Adam optimizer [38] for training.

### 3.2. Quantum-Classical Hybrid Solution

#### 3.2.1. Motivation

To the best of our knowledge, no QML solutions have delved into the domain of deep image AD. To fill this gap, this paper makes an attempt to perform deep image AD with a novel quantum-classical hybrid solution. As DSVDD has been proven to be an effective and pioneering deep AD solution, we naturally chose it as the foundation for developing our hybrid solution. We are interested not only in the feasibility of utilizing QML to address deep image AD, but also in whether our hybrid solution can achieve superior performance to that of its classical counterpart. Our solution is based on the following motivation: We noticed that the regularization term is of vital importance for DSVDD, as it avoids trivially mapping all data to a fixed point in the feature space and losing the ability to discriminate anomalies, i.e. overfitting. Therefore, we assume that the quantum layers can also play a similar role to that of regularization, as the forward pass of the quantum layer requires measurement, which is an inexact step. In this way, we may prevent overfitting of DSVDD and obtain diverse features from input data. In order to verify our assumption, we designed a QHDNN solution for deep image AD and carried out copious experiments to test its performance in terms of various aspects.

#### 3.2.2. The Structure of QHDNN

QHDNN is a hybrid neural network that implements a network with both quantum and classical components. As can be seen in Figure 2, the layers of DSVDD are mainly divided into two parts. The front blue part is mainly responsible for feature learning, i.e., learning features from the raw input data. The later yellow part is mainly responsible for feature mapping and completing the downstream task, which is AD in this case. Considering the different roles of two parts in DSVDD, we mainly explore two possible ways to build an effective QHDNN solution: (a) using a quantum network layer to replace a classical learnable layer in the blue part; (b) using a quantum network layer to replace the fully connected (FC) layer in the yellow part.

Our empirical evaluations suggested that method (b) actually produced better performance than that of method (a) in this case. Specifically, the experimental results showed that the accuracy of method (b) was about 10% higher than that of method (a) (on MNIST). We speculate that such observations can be ascribed to the following reason. Unlike the most frequently seen classical network layers (e.g., the convolution layer) that conduct exact computations and give fixed outputs, the quantum network layer usually includes a measurement process, which is an inexact operation and produces non-fixed outputs. We believe that such an operation can be harmful to the feature learning process in the blue part, but beneficial to the regularization of the final feature mapping process in the yellow part. The intuition behind this is that one usually expects the feature learning layers to extract invariant features from normal single-class data, which can be degraded by the measurement operation in the quantum network layer. By contrast, the inexact property of the quantum layer helps prevent the final feature mapping from trivially mapping all normal data to an identical point in the data space, which causes the loss of generalization ability.

**Figure 3:**
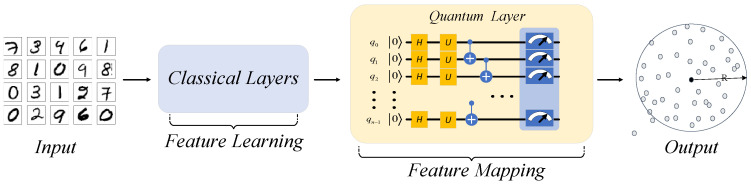
QHDNN. The QHDNN was built by replacing the last FC layer of the classical DNN with the quantum network layer. Therefore, the QHDNN consisted of two parts, a classical feature learning part and a quantum feature mapping part.

Hence, we built our QHDNN solution and conducted further research on the basis of method (b). Figure 3 shows the general structure of our QHDNN. In our architecture, the pooling layers and convolutional layers were kept the same as in a classical DNN, while the quantum layer was adopted to replace the final FC layer for feature mapping.

#### 3.2.3. Design of the Quantum Network Layer

After the overall architecture of the QHDNN was settled, we focused on designing the specific structure of the quantum network layer. Unlike the classical neural network architecture, which has been thoroughly studied, the architecture of the quantum network layer is still being explored. Up to now, the quantum network layer has been implemented in several ways. Some of them do not contain learnable parameters [39], and their performance is often limited due to the lack of learning ability. Recently, an effective quantum network layer containing multiple quantum bits (qubits), which is called the variational quantum circuit (VQC)-based quantum layer [35,40,41,42], has attracted wide attention. It has become one of the most popular quantum layers since it was proposed because it exhibits a comparable learning ability to that of the classical network layer in many cases [43]. We believe that the reason behind its good performance is that VQCs can convert classical vectors into quantum states, and they follow the generalized parameter shift rules [44], which allows them to use a classical optimizer for optimization. Thus, we decided to use a VQC-based quantum layer to build the QHDNN in this article.

A VQC-based quantum layer usually consists of three parts. (a) Preparation: A VQC can only deal with quantum states, but the input of the quantum layer is a classical vector in this case. Thus, before a VQC can fulfill its role, we need to import the classical data into the quantum layer. This can be achieved through a preparation layer P by embedding a real-valued vector x into a corresponding quantum state |x〉. A typical example of this is shown in Equation (4).

(4) P:x→|x〉=E(x)|0〉,

where x is the classical input parameter, |0〉 is the initial quantum state of the qubit that we prepared in advance, and E(x) can be a single qubit rotation or single mode displacement parameterized by x. (b) VQC: A VQC is a circuit comprising a number of quantum embedding layers in a sequential manner. A quantum embedding layer is usually composed of several quantum gates, and some of them are parameterized. A parameterized quantum gate can essentially be viewed as a unitary transformation. Since a quantum state can be written in the form of a vector, a parameterized quantum gate can be expressed as a unitary matrix imposed on the vector. Similarly, a quantum embedding layer composed of quantum gates can also be written as a unitary matrix, which is the product of the unitary matrices representing the quantum gates. This form is shown as follows.

(5) $$U=U_{n}\circ U_{n-1}\ldots \circ U_{1},$$

where $U_{n}$ represents the *n*-th quantum gate in the quantum embedding layer and ∘ represents matrix multiplication. In this way, a parameterized quantum gate can transform one quantum state |x〉 into another output state |y〉, and it is essentially a unitary transformation in a mathematical expression. The functions of these quantum gates are controlled by quantum gate parameters. A VQC layer can be trained by optimizing the parameters associated with the quantum gates. In this way, the quantum layer is able to function like a classical network layer. We can adjust the number of learning parameters by adjusting the number of parameterized gates. Equation (6) shows a normalized expression of the application of a quantum embedding layer on a single qubit, where *U* is a unitary matrix and w is a set of classical parameters.

(6) E:|x〉→|y〉=U(w)|x〉.

Differently from the preparation layer P, the quantum embedding layer E is a mapping of which the input and output are both quantum states. To achieve satisfactory performance in deep AD, we explored a way to enhance the learning capabilities of the quantum network layer. By instinct, we intended to increase the number of learnable parameters in a VQC to accomplish this goal, as this was expected to expand the capacity of the quantum layer. Considering the unique structure of VQCs, we mainly explored two methods: **(1)** increasing the width of VQC, i.e., using more qubits, which required increasing the number of parameterized quantum gates accordingly when building the VQCs. However, the increase in the width of the VQCs would result in the exponential growth of the dimension of the stored state space, which could lead to heavy consumption of computing resources. From another perspective, current noisy intermediate-scale quantum (NISQ) devices [45] cannot support quantum circuits with a large number of qubits. Considering these limitations, this paper limited the numbers of qubits to 8 and 16 in the VQCs. In this way, our QHDNN is computationally feasible for the NISQ scenario while providing enough qubits for our deep image AD task. **(2)** The second method involved increasing the depth of the VQCs, which meant raising the number of quantum embedding layers and stacking them in series. Our experiments showed that this method achieved remarkable performance gains in our experiments, while it did not introduce more qubits or an excessive computational burden. In addition, it was noted that excessive depth could lead to some adverse effects, such as a barren plateau (BP) [46], which refers to a phenomenon in the optimization procedure where the gradients vanish to zero, making the training of VQCs ineffective with gradient-based optimization. Under this circumstance, we cautiously increased the depth of the VQCs to a moderate number (8 to 16). Relevant experimental results are presented in the next chapter. A VQC of depth *n* can be expressed as the product of many parameterized quantum embedding layers, as presented in Equation (7).

(7) $$Q=E_{n}\circ \ldots \circ E_{2}\circ E_{1}.$$

(c) Measurement: Since the outputs of VQCs are quantum states, it is necessary to convert them back into classical data for later layers or tasks. We can define this qubit readout operation as a measurement layer M. Specifically, this layer measures the observable values $\hat{y}$ in a VQC and calculates the expectation of the values for vector y.

(8) $$M:|x\rangle \to y=\langle x\left|\hat{y}\right|x\rangle .$$

As a whole, the quantum layer is still a process in which both the inputs and outputs are classical vectors. This allows the quantum layer to be connected to the rest of the classical parts. The complete quantum layer can be represented as:

(9) Q=M∘Q∘P.

**Figure 4:**
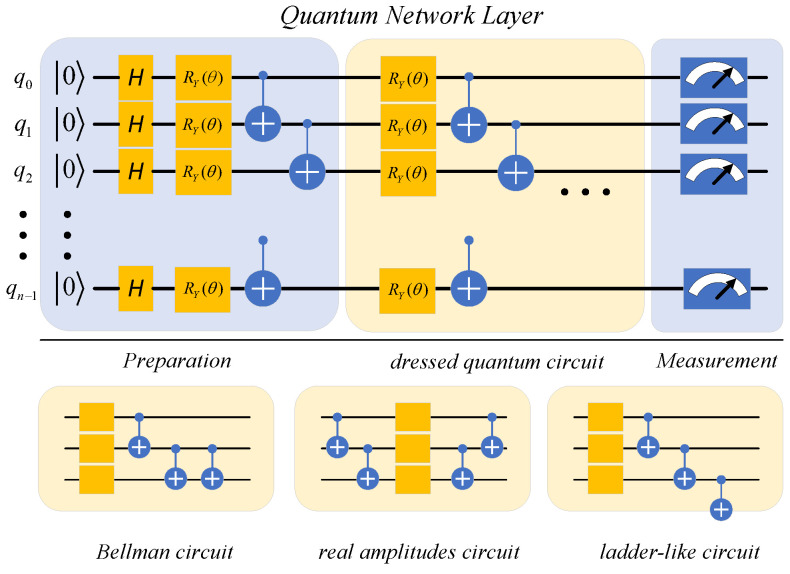
Schematic representation of the quantum network layer. The preparation layer converts a real vector into a quantum state. The yellow part is a VQC of depth *n* that contains learnable parameters. The measurement layer converts quantum states back into classical data for later layers or downstream tasks.

We also carried out experiments on different VQC structures with the goal of finding the most suitable structure for deep AD. The experimental results indicated that different structures of VQCs could lead to significantly different results, and DC provided better performance than that of other structures. Therefore, as shown in Figure 4, we decided to design our quantum layer on the basis of DC.

## 4. Experiments

In this section, we describe our experimental setup, benchmark datasets, and evaluation metrics. We conducted extensive experiments on typical image benchmarks to test our hybrid QML solution. Finally, we show the results of testing our QHDNN on Rigetti’s quantum SDK Forest.

### 4.1. Settings of Experiments

To verify the feasibility of QML in solving deep image AD, we compared the performance of our hybrid solution with that of its classical counterpart. The experiments were mainly conducted on two frequently used benchmark datasets: MNIST and FashionMNIST. MNIST is a dataset that consists of 70,000 28×28 gray handwritten images of 10 digits, with 7000 per class. There are 60,000 training images and 10,000 test images, which are divided equally across the classes. FashionMNIST is a relatively new dataset with 28×28 grayscale images, which also include 70,000 fashion products from 10 categories, with 7000 per category. The training set has 60,000 images, and the test set has 10,000 images.

We adopted the one-against-all evaluation protocol in our experiments, which is widely used in deep AD. Specifically, for 1≤M≤10, we fetched 6000 images of the class *M* in the original training set as a new AD training set $S_{M}$, and all images were viewed as normal during training. Then, we still used the whole test set of 10,000 images for testing, but it was re-labeled by specifying images of class *M* as normal and others as abnormal. Thus, we could conduct 10 different experiments by alternatively using data from different classes as the normal training set. As the training set $S_{M}$ only contained normal images (belonging to class *M*), the QHDNN intended to learn a hypersphere based on it. The hypersphere was then applied to the test set, which contained both abnormal (not class *M*) and normal samples (class *M*) to evaluate the AD performance of the network. We quantitatively evaluated the results with the area under the receiver operating characteristic (AUROC) [47], which is called AUC in the rest of our paper for simplicity. Due to the computational cost of quantum measurement, we controlled the size of $S_{M}$ to keep it relatively small by randomly sampling 400 images from the original $S_{M}$. For a reasonable and fair comparison, in our experiments, the learnable parameters of our QHDNN were set to be identical to those of the classical DNN in DSVDD. Specifically, the number of learnable parameters in the final FC layer of the classical DSVDD ($N_{c}$) was set to be consistent with the parameter number of the quantum layer in QHDNN ($N_{q}$) when performing the experimental comparison. In our experiments, we tested 64 and 256 as two typical values for $N_{c}$ and $N_{q}$.

### 4.2. Results and Discussion

#### 4.2.1. Results

In Table 1, we present our main results. The overall AUC of each solution is shown; this is the average of the AUCs obtained in 10 different experiments (using classes 0 to 9 alternatively as the normal class). In the table, “AUC” denotes the results obtained by training the model with a fixed number of epochs (epochs = 10), while “AUC*” denotes the best model performance when the training epochs were varied in a range. Some interesting observations can be found in Table 1. **(1)** Notably, when sharing the same number of learnable parameters ($N_{c}=N_{q}$), our hybrid QHDNN solution achieved comparable results to those of the classical DSVDD solution, and even outperformed it in most cases. For example, on MNIST, an 86.273% AUC could be achieved with the classical DNN with $N_{c}=256$ and a training set size of 300. By contrast, our QHDNN was able to achieve an 88.237% AUC under the same conditions, which was 1.9% higher than the score of its counterpart. Similar phenomena could also be observed for FashionMNIST. For instance, when the number of parameters was set to 64, the QHDNN achieved an AUC of 87.591% on FashionMNIST, which was 1.5% higher than the result of the classical DNN. **(2)** We could see that the increase in learnable parameters tended to improve the AUCs of both the QHDNN and the classical DNN, while the QHDNN outperformed the classical DNN in most experiments. For instance, when the number of parameters was raised from 64 to 256, the AUC of the QHDNN on MNIST increased from 87.116% to 88.237%, while the classical DNN of DSVDD grew from 85.854% to 86.273%, and the QHDNN maintained its superior role in comparison. The situation was similar for FashionMNIST. However, when the number of epochs was fixed to 10, the classical DNN performed marginally better than the QHDNN. Note that the performance of the classical DNN was slightly inferior to that in the original work [21], as $N_{q}$ could not be too large due to characteristics of NISQ, which also limited $N_{c}$ in comparison.

#### 4.2.2. Discussion

**Figure 5:**
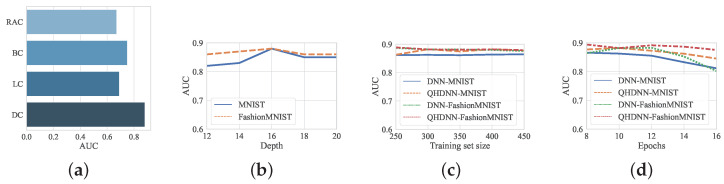
(**a**) Results with different structures of VQCs. (**b**) Results when the depth of VQCs varied. (**c**) Results when the training set size changed. (**d**) Results when the number of epochs changed.

**Qubits and Depth:** As mentioned in Section 3.2.3, we chose the depth (D) of the VQCs and the number of qubits from a certain range. Figure 5b shows the change in the performance with different values of D. It can be seen that when D varied between 12 and 20, the AUC of our QHDNN remained satisfactory (above 80%) in all cases. Interestingly, the optimal results could be achieved on both MNIST and FashionMNIST when D was set to 16. When D exceeded 16, the AUC of our hybrid solution began to drop. This performance loss could be attributed to the overfitting caused by excessive learnable parameters. In the following discussions, we set the number of qubits and D to 16 by default.

**Different VQC Structures:** We conducted experiments on the real amplitude circuit (RAC) [48], the Bellman circuit (BC) [48], the ladder-like circuit (LC) [49], and the dressed quantum circuit (DC) [35]. The experimental results are displayed in Figure 5a. The outcome indicated that different structures of VQCs could lead to different results, and the DC provided significantly better performance than that of the other structures. We speculate that this was because the quantum entanglement process of the DC performed better than the other structures, which was due to the fact that its entanglement gates were alternately arranged on the adjacent qubits. This made the entanglement of the entire circuit relatively stable.

**Training Set Size and Epochs:** The size of the experimental training set and the number of training epochs may also have affected the performance of our solution. As presented in Figure 5c, our QHDNN achieved good performance when the size of the training set was varied from 250 to 450, and it showed high stability on both MNIST and FashionMNIST. As shown in Figure 5d, when the number of training epochs was changed from 8 to 16, the performance of the QHDNN could be maintained at a good level. However, when the number of epochs was further increases beyond 12, the AUCs of both the QHDNN and the classical DNN started to decline, which was probably due to the overfitting problem. Interestingly, we found that the performance of the hybrid method was more robust than that of its counterpart.

**Quantum Virtual Machine (QVM):** The above experiments were based on a simulation with a classical computer, and the simulation results validated the effectiveness of our QHDNN. In order to realize a more realistic simulation of the quantum circuits in our QHDNN, we also conducted experiments on Rigetti’s Forest SDK [50] to test our hybrid solution. The Forest SDK contains the Quantum Virtual Machine (QVM) and the quilc quantum compiler. The QVM could fully simulate how the QHDNN would behave on a real quantum computer, since it can simulate the noise generated by a real quantum device. In our experiments, we compared the performance of our previous simulations with that of the simulations on the QVM. As an example, we tested our QHDNN with 8 qubits and 64 learnable parameters on the QVM, and the results showed that the AUC of our hybrid solution was only slightly worse (within 2%) when compared with our previous simulations, which indicated the feasibility of our hybrid QML solution for working in the presence of noise.

### 4.3. Conclusions and Future Work

In this paper, we presented a QHDNN that was used to solve deep image AD problems. Based on DSVDD, our QHDNN can learn directly from raw input image data to train a normality model and then classify the data that do not fit the model as anomalies in its inference. In the process of building the QHDNN, we explored various methods of designing the quantum layer and designing an optimal architecture. We then conducted extensive experiments on two commonly used benchmarks to test our hybrid solution. According to the results of the experiment, it was shown that with the same experimental settings, our QHDNN achieved comparable or even better results than those of the classical DNN. In addition, we also tested our hybrid solution on Rigetti’s QVM, which justified our QHDNN’s ability to tolerate noise.

The contribution of our study is not limited to the improvement in the accuracy in deep AD, but moreover, our work also leaves some interesting directions for future research. (1) Although our solution is based upon the DSVDD framework, the QHDNN can also be designed based on other deep AD solutions, such as that of [30], which may produce further performance escalations. (2) Due to the limits on the qubit number and the gradient vanishing problem, the learnable parameters of the quantum layer cannot be too numerous. This will be meaningful when searching for a better VQC architecture so as to further enhance the QHDNN’s performance by increasing the number of parameters. (3) It would be interesting to implement our hybrid solution on real quantum devices in the future. 

## Figures and Tables

**Table 1:** Main results. “$N_{q}/N_{c}$” represents the number of learnable parameters of the quantum layer in the QHDNN or parameters of the corresponding layer in DSVDD. “AUC” denotes the results obtained with a fixed number of epochs (epochs = 10), while “AUC*” denotes the best model performance when the training epochs were varied in a range.

Dataset	Network Type	Training Set Size	Qubit	Depth (D)	$N_{q}/N_{c}$	AUC	AUC*
MNIST	DNN	300	0	0	64	85.854%	86.754%
	QHDNN	300	8	8	64	**87.116%**	**87.116%**
	DNN	300	0	0	256	86.273%	86.588%
	QHDNN	300	16	16	256	**88.237%**	**88.237%**
FashionMNIST	DNN	300	0	0	64	86.032%	86.725%
	QHDNN	300	8	8	64	**87.591%**	**88.132%**
	DNN	300	0	0	256	**88.201%**	88.292%
	QHDNN	300	16	16	256	88.186%	**89.414%**

## Data Availability

The raw/processed data will be made available upon request.
